# Using structural diversity to measure the complexity of technologies

**DOI:** 10.1371/journal.pone.0216856

**Published:** 2019-05-21

**Authors:** Tom Broekel

**Affiliations:** Department of Human Geography and Spatial Planning, Faculty of Geosciences, Utrecht University, Utrecht, The Netherlands; Northwest University, UNITED STATES

## Abstract

The paper introduces structural diversity as a new approach to quantify the complexity of technologies. By modeling technologies as combinatorial networks, a measure of technological complexity is derived that represents the diversity of (sub-)network topologies in these networks. It is further argued that this measure can be empirically approximated with the Network Diversity Score (NDS). The paper also presents an application of this approach to European patent data from 1980 to 2015. On this basis, the measure of structural diversity is shown to replicate a number of stylized facts commonly associated with technological complexity: Complexity increases over time and younger technologies are more complex than older technologies. Complex technologies are also associated to larger R&D efforts and require more collaborative R&D activities. Lastly, when controlling for technologies’ size, technologies scoring high on structural diversity are also shown to concentrate in space.

## 1 Introduction

### 1.1 Measuring the complexity of knowledge

The complexity of knowledge is seen as a crucial explanatory dimension of technological development and economic success [1–5]. The higher difficulty of inventing and learning complex knowledge is argued to require larger economic efforts of entering these domains. This hinders the diffusion of such knowledge among economic agents [6, 7]. Consequently, complex knowledge can be expected to be more exclusive and therefore to possess more economic value [8, 9].

However, empirical studies analyzing technological and innovation processes frequently rely on simple counts of knowledge inputs or outputs (e.g., patents, number of new products), and thereby fail to capture this dimension of knowledge [5]. This shortcoming seems to be less a matter of recognizing the dimension’s importance and more of a lack of a convincing (quantitative) measure of knowledge complexity [5]. There have been many attempts in different disciplines to measure knowledge complexity [5, 10–15]. However, for most of these, it is still unknown if and if so how they are applicable to real world data as well as whether they allow for differentiating knowledge domains according to degrees of complexity.

Two notable exceptions in this respect are the works of *Fleming and Sorenson* [13] and *Balland and Rigby* [5]. Based on an N/K model, *Fleming and Sorenson* quantify the degree of interdependence inherent to subcomponents of a knowledge domain, which these authors interpret as an approximation of knowledge complexity. The applicability of this approach to patent data is demonstrated in multiple empirical studies [7, 16]. However, to the best of the author’s knowledge, *Fleming and Sorenson*’s measure has not been used to evaluate complexity at the level of technologies. Nevertheless, in principle, its patent-specific complexity values can easily be aggregated to the level of technologies. In contrast, *Balland and Rigby* [5] apply *Hidalgo and Hausmmann’s* *economic complexity index*, which was originally designed to assess the complexity of countries’ export and employment patterns, to patent data, and thereby obtain an index of knowledge complexity [4, 5].

However, both approaches rely on strong assumptions. The complexity measure of *Fleming and Sorenson* is build on the idea that subcomponents of complex inventions are difficult to combine, which translates into such combinations being relatively infrequent [13]. Yet, economic reasons unrelated to difficulties in inventive processes might influence the frequency of knowledge combinations. For instance, the lack of some combinations’ market potential can result in minimal attention from researchers. Alternatively, as noted in other works of these authors, it may instead be the range of applications shaping the combinatorial frequency, which may or may not reflect complexity [7].

The index of *Balland and Rigby* rests on the assumptions that complex knowledge is relatively scarce geographically and that it tends to co-concentrate with other complex knowledge in space [5]. However, the spatial distribution of knowledge may have multiple explanations, including complexity. For instance, the diffusion of knowledge in space and, hence, its geographic distribution, depend on its degree of maturity, popularity, natural conditions, geographic distance, place of origin, and (again), crucially, economic potential [17–20]. From an empirical perspective, constructing a complexity index on the basis of the spatial distribution of knowledge raises two additional issues: It represents a potentially endogenous variable in many spatial research settings and its values are conditional on the delineation of the employed spatial units.

Lacking an objective criterion of knowledge complexity, it is difficult to assess the severity of these assumptions. Hence, the extent to which these measures actually capture what they are intended to capture is unclear.

The present paper contributes to the literature with a novel approach to this matter. Based on a conception of technologies as combinations of (knowledge) components [13, 21], it uses insights from complexity and network science to introduce *structural diversity* as a new measure of technological complexity. It represents the diversity of (subnetwork) topologies in technologies’ combinatorial networks. The paper also puts forward that the Network Diversity Score developed by *Emmert-Streib and Dehmer* [22] to quantify the complexity of networks, can serve as an empirical approximation of this measure.

Using this approach, the complexity of 655 technologies is quantified on the basis of EU patent data from 1980 to 2015. The results are shown to correspond to a number of stylized facts, which the literature suggests characterize technological complexity: Complexity is increasing over time, it requires more R&D and it involves more collaboration. In contrast, the stylized fact of complex technologies to concentrate in space is only confirmed when controlling for the size of technologies.

### 1.2 Technologies as combinatorial networks

In management science and engineering, *technologies* are described as systems of interrelated components [21, 23, 24]. Components are all “*parts of a technology or the steps in an industrial process*” (p. 9009) [24], and two components are related if changes in one of them affects the respective other. A comparable conceptualization can be found in innovation studies. In this literature, *technologies* are described as sets of interrelated components with the latter being knowledge “pieces” and relations being their combinations [16]. For example, “*one might think of the automobile as a combination of the bicycle, the horse carriage, and the internal combustion engine*” (p. 1020) [13]. While the conceptualization in engineering is instead focused on technological systems, with the set of relations between components being known as *design structure matrix* [25], innovation studies focus on the knowledge dimension of technologies. In this literature, a technology is described as a *recipe*, which encompasses information on constituent knowledge components and their combinations [16]. Crucially, both views apply a network perspective with nodes representing components and links representing their relations/combinations. In these networks, some components are directly linked while others are indirectly related. For instance, in regard to airplane technology, the components’ wing design and aluminum processing are directly linked, while electronic navigation is only indirectly related since other components (e.g., electronic control systems, mechatronic interfaces) act as bridges.

The network perspective allows the assessment of technologies’ complexity based on the combination of their components [13, 16, 24]. The measure of *structural diversity* extends this approach with insights from network science. In such research, a wide range of measures has been put forward that quantify the structural complexity of networks [26, 27]. Importantly, some measures evaluate networks’ complexity from the perspective of information theory [28, 29]. In essence, information theoretical measures of network complexity quantify the amount of information contained in networks, i.e. the quantity of information needed to describe the full network, or alternatively to describe its most important structural features (e.g., their degree distribution). In any case, a network’s complexity is argued to increase with growing amounts of information needed for its description [30].

**Fig 1:**
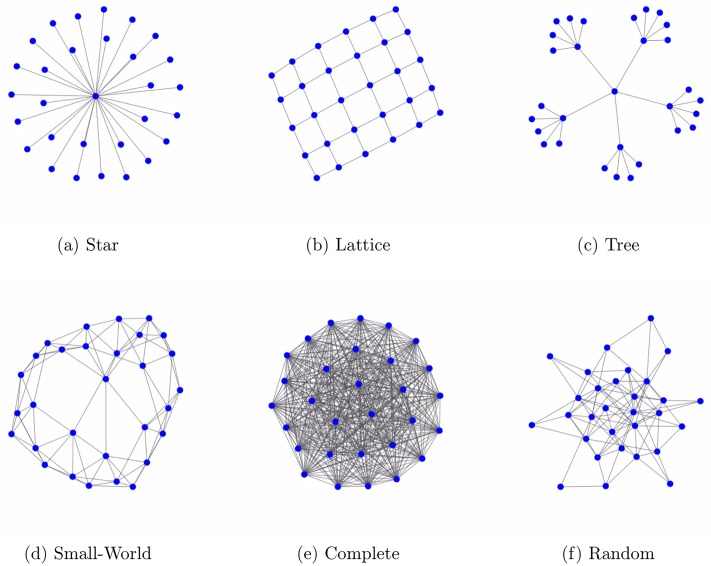
Archetypal network topologies.

Lets assume a technology’s combinatorial network has a star-like structure (Fig 1a). This network, and thereby the technology, can be easily described once this structure and the central knowledge component are known. In this case, the technology only consists of this central knowledge component and its direct combination with all other components. From an information theoretical perspective, the description of this technology requires minimal information because there is only one (simple) network structure dominating its combinatorial network. The low information content makes it likely that this technology can be easily invented, copied, and codified. An (oversimplified) example of such a technology with a star-like combinatorial network is a table. It usually consists of five components (four poles and one table board). Each pole is directly and exclusively connected to the table board, which therefore constitutes the central component. From the information theoretical perspective, the combinatorial network of the table indicates it is a simple technology.

Similarly, only a limited amount of information is required to describe a lattice network (Fig 1b), as its degree distribution already contains most of its structural characteristics. More information is contained in a tree-like network (Fig 1c). Here, in order to be described, the identity of multiple central nodes and the depth of its hierarchy are needed. The amount of information to represent a small-world network (Fig 1d) is even larger. In fact, small-world structures are usually used as examples for complex networks [22].

What differentiates simple networks from more complex ones is the existence of certain kinds of organizational principles underlying their structures. These principles allow for condensing the information required to describe a network. Usually, these structuring principles are the result of specific network formation mechanisms, such as preferential attachment or transitivity. The existence of organizational principles tends to translate into specific (sub)network structures (e.g., stars, lattices, cliques, hierarchies) that appear in a network. These are called network *topologies* in the remainder of the paper, and they are the basis for the proposed approach for measuring technological complexity.

In line with an information theoretical view on network complexity, I argue that the more information is required to describe the topology of a technology’s combinatorial network, the more complex it is. Moreover, multiple topologies will usually be needed to describe a combinatorial network’s structure, as these networks may consists of subnetworks, each with different topologies. In other words, it is unlikely that a technology’s components will connect in just one way (e.g., in a star-like manner). Alternatively, topologies may also be mingled. For instance, a small-world network combines tree-like and clique-like topologies. Since each topology’s description implies additional information, the total information required to describe the full network will increase the more distinct topologies are present. With increasing information, the complexity of the combinatorial network grows along with the complexity of the corresponding technology. Consequently, a quantification of this diversity in topologies can be seen as a measure of technological complexity, which will be called *structural diversity* in the following. Notably, according to this view, a random network (Fig 1f) is the most complex theoretically because it consists of the largest number of distinct topologies.

## 2 Method

### 2.1 The measure of structural diversity

There is no commonly accepted method of assessing the complexity of networks or the diversity in its sub-structures and topologies. It is beyond the scope of the present paper to review or discuss the pros and cons of the many existing approaches, as this can be found elsewhere [22, 26, 27].

Recently, the *Network Diversity Score* (NDS) was introduced and compared to other common measures of network complexity [22]. In contrast to these measures, only the NDS is capable of consistently separating ordered, complex, and random networks. Networks are considered *ordered* when many nodes exhibit similar properties (e.g., degree), which corresponds to one or a few dominant topologies. According to the previous discussion, ordered combinatorial networks represent relatively simple technologies because of their comparatively more homogeneous topologies. *Complex* networks represent mixtures of *ordered* and *random* structures. They are therefore characterized by a larger topological heterogeneity compared to *ordered* networks. For example, a small-world network is usually seen as complex [22] because it involves multiple topologies such as stars and triangles of different sizes (Fig 1d). Random networks are the most structurally diverse as they involve the largest heterogeneity of topologies. Despite the variance in specific network topologies used in its definition, the NDS does not directly measure the structural diversity of networks. However, it ranks networks on a scale ranging from ordered over complex and to random networks, which empirically represents the idea of *structural diversity*. Crucially, this does not suggest that technologies with random combinatorial networks actually exist or are even possible. Ultimately, the cumulative character of technological development and the relevance of social processes underlying it will ensure the presence of systematic structures.

The NDS differs in multiple ways from traditional measures of network complexity. Firstly, it is a result of scientific numerical experimentation. More precisely, while based on some general theoretical ideas on what characterizes simple and complex networks, the measure is empirically optimized to significantly differentiate between artificially created random, ordered, and complex networks [22]. Secondly, the measure combines multiple network characteristics into one: It considers the share of modules ($\alpha_{module}=\frac{M}{n}$) with *M* being the number of modules and *n* being the number of nodes. Modules are densely connected subgraphs in a network. The variance of module sizes *m* $v_{module}=\frac{var(m)}{mean(m)}$ is also included. Random networks are likely to show a low variability and low average size of modules. Further, the variable *V*_λ_ captures the Laplacian (*L*) matrix’s variability defined as $v_{\lambda }=\frac{var(\Lambda (L))}{mean(\Lambda (L))}$. Lastly, the relation of motifs of sizes three and four enters the measure. This variable is observed to be highest in ordered networks, medium in complex networks, and lowest in random networks [22]. Counting the number of motifs in networks usually implies concentrating on those network three- and four-node structures that are overrepresented in the empirical network in comparison to a random network [31]. Due to the substantial computational burden of the randomization of all sample networks, I adapt this part of the NDS measure and replace the motifs-based relation with the ratio of graphlets of sizes three and four. Hence, I estimate the ratio *r*_*graphlet*_ between all empirically observed network structures based on three nodes (graphlets of size three, *N*_*graphlet*_(3)) and those involving four nodes (graphlets of size four, *N*_*graphlet*_(4)) as $r_{graphlet}=\frac{N_{graphlet}\left(3\right)}{N_{graphlet}\left(4\right)}$.

The four variables are combined in the individual network diversity score (*iNDS*) of the network (*G*_*T*_):

$$\begin{array}{c} iNDS\left(G_{T}\right)=\frac{\alpha_{module}*r_{graphlet}}{v_{module}*v_{\lambda }}. \end{array}$$ (1)

Networks may show properties of a complex or ordered network merely by chance and thereby mislead measures of complexity. Therefore, *iNDS* is estimated for a population of networks *G*_*M*_ to which *G*_*T*_ belongs [22]. In practice, drawing random samples *S* from network *G*_*T*_ and estimating *iNDS* for each sample network achieves this. The final network diversity measure (*NDS*) is obtained by:

$$\begin{array}{c} NDS\left(\left\{G_{T}^{S}|G_{M}\right\}\right)=\frac{1}{S}\sum_{G_{T}\epsilon G_{M}}^{S}iNDS\left(G_{T}\right) \end{array}$$ (2)

To allow for an easier interpretation, I transform the measure such that large values signal random networks (complex technologies), medium values indicate complex networks (medium complex technologies), and low values represent ordered networks (simple technologies). This is done by taking *NDS* in logs and subsequently multiplying it by −1. The obtained value represents the structural diversity of a technology’s combinatorial network and will be denoted as *structural diversity* in the remainder of the paper. Notably, its values may vary somewhat when estimating it repeatedly for the same technology due to the random sample selection procedure.

### 2.2 Data

Using the measure of *structural diversity* I estimate the complexity of technologies on the basis of patent data. Despite well-known problems [32], patents entail detailed and unparalleled information about technologies and their innovation processes. I use the OECD REGPAT database (version 2018) covering patent applications to the European Patent Office. As there is a time lag between the priority date and the availability of patent information, the most recent years of this data are unreliable. The analysis is therefore restricted to the years 1980 to 2015. It utilizes information on 3, 137, 881 patent applications. They are assigned to countries and regions by means of inventors’ residences (multiple-counting). Technologies are defined on the basis of the *Corporate Patent Classification* (CPC). The CPC is hierarchically organized into nine classes at the highest level and into more than 230,300 subclasses at the lowest level. I use the four-digit CPC level to define 655 distinct technologies. While there is no objective reason for this level, it offers a good trade-off between technological disaggregation and manageable numbers of technologies. In addition, it has been used in related studies [33, 34].

Patent numbers vary considerably between years and some technologies have few patents. Therefore, a moving window approach is used to calculate annual complexity measures. In other words, I combine the patent information of three years such that a technology’s complexity measure in year *t* is based on patents issued between years *t* and *t* − 2. The choice of three years is admittedly rather arbitrary. However, it represents a good trade-off between the smoothness of the development and the temporal variance of the measure.

### 2.3 Calculating structural diversity

When applying the structural diversity measure to patent data, technologies’ (knowledge) components and their combinations must be defined. I consider the lowest level of CPC classes (10-digit subclasses) as approximations of components and their co-occurrence on patents as combinations [13, 16]. The estimation is done on this basis as follows for each year *t* (moving window of three years): First, for each of the 655 technologies *T*, all patents are extracted with at least one of their 10-digits CPC subclasses belonging to the four-digit class of the focal technology (*Pats*_*T*_). Second, the matrix *M*_*T*_ is created from all co-occurrence counts of the (ten-digit) CPC subclasses assigned to the patents *Pats*_*T*_. *M*_*T*_ is dichotomized with all positive entries set to one. The dichotomization is necessary because the *NDS* measure is not (yet) defined for valued networks. While the dichotomization keeps most of the original (valued) network’s structural information, it still implies a substantial simplification, which needs to be addressed by future work.

*M*_*t*_ is an adjacency matrix representing the network *G*_*T*_ of all of the ways technology *T*’s components have been combined among themselves, how they have been combined with other technologies’ components, and how other technologies’ components are combined when at least one component of technology *T* is involved. Hence, it summarizes all (knowledge) combinations related to technology *T*, i.e. a technology’s combinatorial network. Alternatively, the network can be restricted to component combinations exclusively involving technology *T*. However, I refrain from this because such a restriction would ignore potential bridging functions of adjacent technologies and how technology *T* is embedded into the overarching technological space.

The *structural diversity* of technology *T* is obtained by applying the *NDS* measure to network *G*_*T*_. However, as the *NDS* requires connected networks [22], the estimation is restricted to the main component of network *G*_*T*_. While in early years (<1985), the largest component represents less than 50% of the combinatorial networks’ nodes, its size rises quickly and on average represents more than 75% of the nodes by 1997 (see S2 Fig in the supporting information). For each *G*_*T*_ (main component), a set of *S* nodes is randomly selected. For each node *c* (*c ϵ S*), a network *G*_*T*,*c*_ is drawn from *G*_*T*_ by a random walktrap of *n* steps starting from *c*. In case the network has fewer than *S* nodes, *S* was set to its number of nodes. The choice of *S* and *n* represent a trade-off between robustness and computational burden. Previous work found a sample size of *S* = 10 and *n* = 120 to be sufficiently robust for comparable real-world networks [22]. Inspired by this, *S* is set to 50 and *n* to 150 in this paper. The *iNDS* (Eq 1) is then calculated for all sample networks *G*_*T*,*c*_ and subsequently averaged (Eq 2). The results is denoted as *NDS*_*T*_ and it represents the empirical measure of *structural diversity* of the combinatorial network of technology *T*. The measure is separately calculated for each technology in every year *t* resulting in a year and technology specific complexity value. That is, the multi-step procedure of calculating *NDS*_*T*_ is repeated 655 × 38 (technologies × years) times.

## 3 Empirical analysis

The presentation of the empirical results is centered on four stylized facts of technological complexity that most scholars in the field seem to agree upon: Technological complexity increases over time, complex technologies involve more R&D and require more collaboration. Moreover, complex technologies tend to concentrate in space.

### 3.1 Technological complexity increases over time

**Fig 2:**
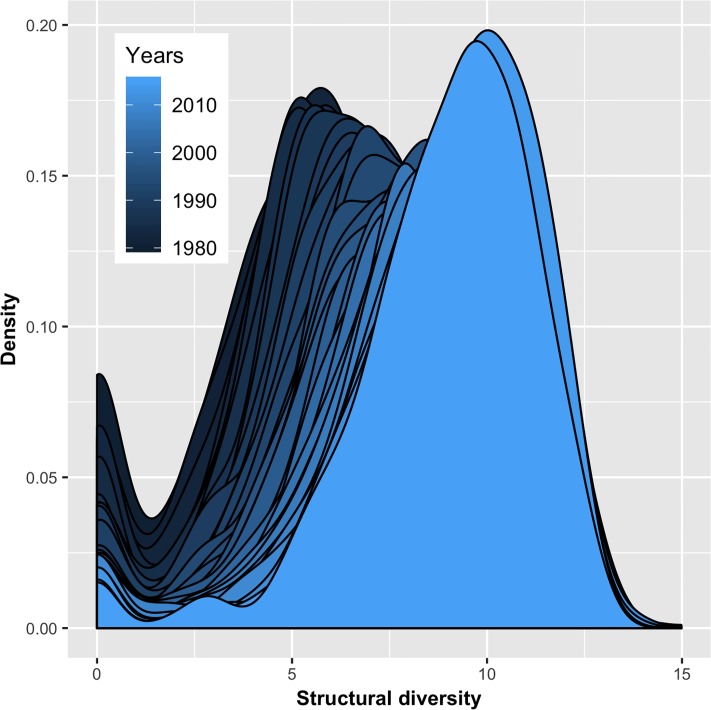
Distribution of structural diversity 1980-2015.

Fig 2 presents the distribution of *structural diversity* of the 655 technologies in each year between 1980 and 2015. The observed minimum is zero and the maximum is 14.98. In between, the distribution is bimodal with a peak at zero and a maximum density at moderate values. The peak at zero reflects many technologies having no or too few patents to calculate *structural diversity* because the main component of the combinatorial needs needs to be at least of size two for the calculation of the NDS. Due to generally rising patent numbers, this peak becomes less pronounced over the years. When abstracting from the peak at zero, the distribution is bell-shaped with short right-hand and somewhat longer as well as over time growing left-hand tails. Otherwise, the general shape remains relatively similar over time. The relative stability of technologies’ rankings is confirmed by the measure’s temporal (rank) correlation (S1 Fig in the supporting information). With few exceptions, the median increases over the years, which gives a first impression of *structural diversities’* temporal development. Technological complexity is argued to increase over time due to knowledge and technologies’ cumulative natures, thus implying that each generation is building upon the technological environment established by its predecessors [35–38]. Technologies also become more complex due to their growing range of functions. For instance, “[d]*igital control systems* [of aircraft engines] *interact with and govern a larger (and increasing) number of engine components than* [previous] *hydromechanical ones*” [p. 904] [39]. Another example is Microsoft’s operation system Windows, which grew from 3-4 million lines of code (Windows 3.1) to more than 40 million (Windows Vista) [40]. Moreover, technologies have reached higher levels of complementarity requiring more multi-technology activities, which adds to the complexity of their development and application [41]. In summary, “[t]*he result is a constantly increasing sophistication and richness of the technological world*” (p. 773) [37].

**Fig 3:**
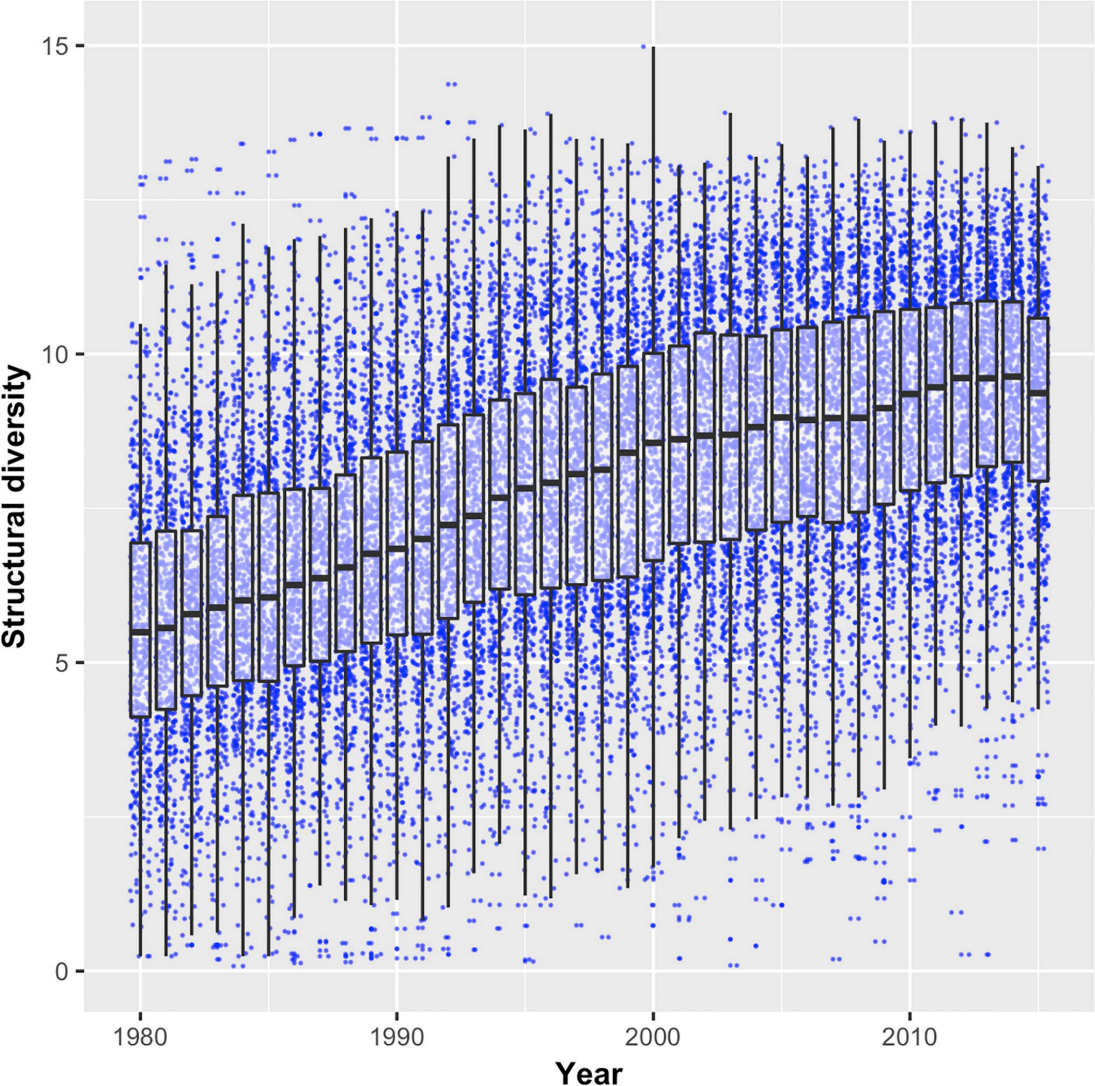
Development of structural diversity over time (1980-2015).

The boxplots in Fig 3 reveal the median *structural diversity* of the 655 technologies to grow over time, which is in line with the argument of increasing technological complexity (a jitter algorithm has been used to distribute the dots for maximizing visibility). Notably, the variance of *structural diversity* remains high with the lowest values observed for 2015 being well below the median in 1980. Moreover, a set of technologies already reaches values in the early 1980s larger than the highest values in most recent years. However, very low patent numbers characterize these technologies, which makes the patent-based assessment of their technological complexity less reliable.

**Fig 4:**
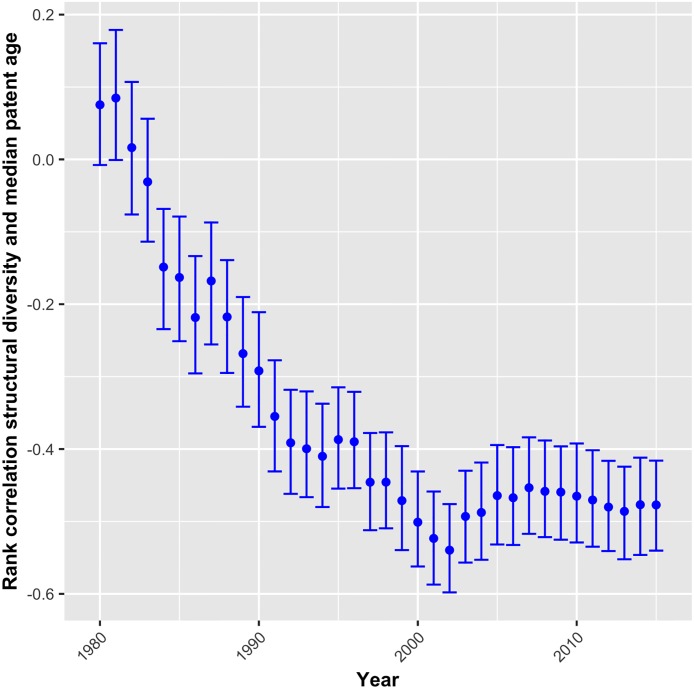
Correlation between structural diversity and technologies’ age, 1980-2015.

Another way to examine the evolution of complexity over time is to compare young and old technologies. For this comparison, the age of all patents has been calculated by subtracting their priority year from the most recent year in the data (2015). A technology’s age in year *t* is then represented by the median of the age of all patents (at least one of their CPC subclass belongs to this technology) that have been granted in *t* or before. The rank correlation coefficient of technologies’ *structural diversity* value and their patents’ median age are plotted in Fig 4 for each year. With the exception of 1980 to 1983, the correlation is significantly negative, thus signaling that younger technologies obtain higher values of *structural diversity*. From 1992 onwards, this correlation is very strong, with the coefficient fluctuating around *r* = −0.47. The finding implies that the increase of technological complexity over time is partly explained by new technologies being more complex than older ones.

### 3.2 Complex technologies require larger R&D efforts

Technologies are advanced by creating new knowledge combinations through search activities for potentially fitting pieces and subsequent testing of these combinations, which is frequently done by trial-and-error [42]. “Harder-to-find,” i.e. more difficult/complex solutions, involve more trials and errors, which consume resources. Complex technologies are based on greater knowledge diversity and on the combination of less common knowledge than simple technologies [13], which further increases the efforts needed in development processes. Additionally, learning complex knowledge is more resource-intensive because greater absorptive capacities are needed [43] and passive learning modes are insufficient [14]. These features of complex technologies translate into longer development times for complex products [44]. In line with this, organizations are more likely to fail when engaged in the development of complex technologies [45]. At the national level, R&D intensity is moreover observed to outgrow economic outputs and incomes because of increasing complexity and development diversity [14, 46]. In sum, the development of complex technologies requires more R&D efforts than simpler technologies.

Unfortunately, there is hardly any information on R&D efforts available that can be matched to the employed patent data. I therefore use two alternative approximations, none of which is perfect: patents and being classified as high-tech. Patents and R&D efforts are positively correlated at the organizational and regional levels [32, 47] suggesting that total R&D efforts are larger in technologies with many patent applications. However, there are also considerable differences in industries’ patent propensities [48], which may distort this relation.

**Fig 5:**
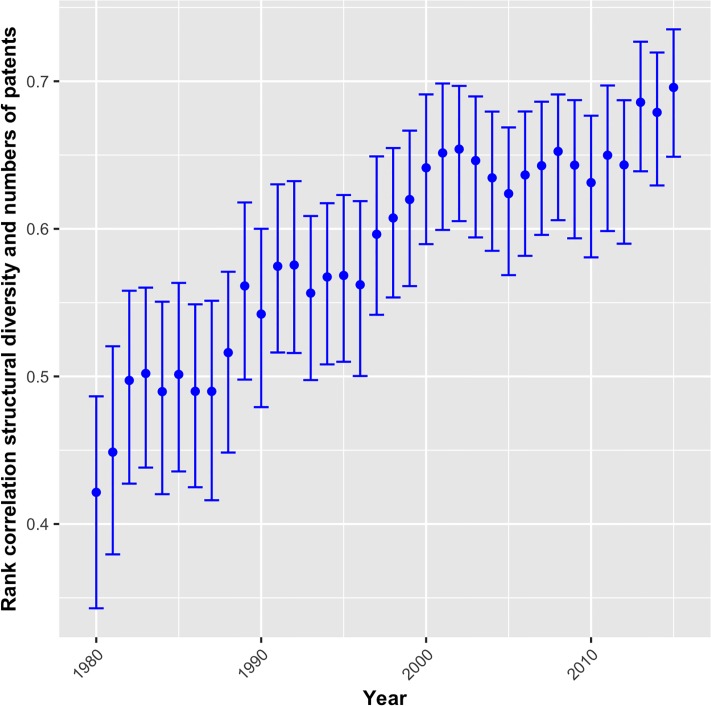
Correlation between structural diversity and technologies’ patent counts, 1980-2015.

Fig 5 shows the rank correlation coefficients for the years 1980 to 2015 of *structural diversity* and the number of patents assigned to a technology. The correlation coefficient is strongly positive and significant in all time periods ranging from *r* = 0.45 (1980) to *r* = 0.69 (2015). To the extent that patent numbers reflect R&D intensity, it confirms the positive relation between complexity and R&D intensity. The positive trend in the coefficient’s development suggests that this relationship intensifies over time—i.e. reaching higher levels of complexity is more dependent on R&D investment than in the past. Potentially, this trend reflects the diminishing returns to R&D hypothesis according to which innovations are increasingly distributed across more products, which results in declining returns to R&D over time [49].

As an alternative measure of R&D efforts, I compare high-tech and non high-tech technologies. High-tech research is characterized (and frequently defined) by larger R&D efforts and intensity [50]. It is also directly linked to complex technologies [51, 52]. Accordingly, technologies considered high-tech are expected to obtain larger values of *structural diversity* than other technologies.

The Trilateral Statistical Report from the European, Japanese, and US patent offices identifies 31 four-digit patent subclasses as high-tech [53]. High technologies include the fields of computer and automated business equipment, aviation, microorganism and genetic engineering, lasers, semiconductors, and communication.

**Fig 6:**
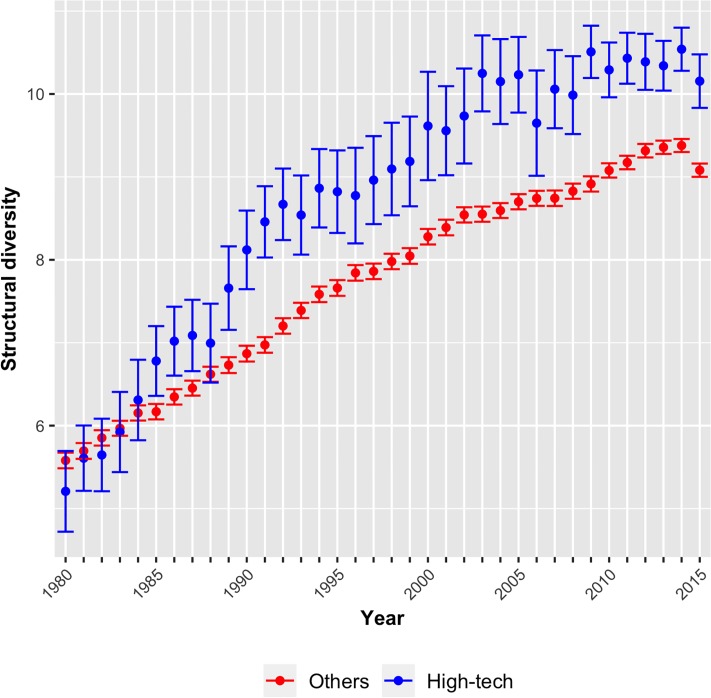
Structural complexity of *high-technologies*, 1980-2015.

Fig 6 compares the *structural diversity* of high technologies with all other technologies. With the exception of one year (1981), high technologies show on average larger values of *structural diversity*. The difference becomes statistically significant in the late 1980s. Hence, the measure of *structural diversity* also identifies high technologies as more complex, which provides further support for the argument that complex technologies require larger R&D efforts. Nevertheless, these results should not be over-interpreted because patent counts and belonging to high technology are far from being precise approximations of R&D intensity.

### 3.3 Complex technologies require more collaboration

Another feature commonly associated with complex technologies is their greater need for collaboration in R&D [4, 5, 38]. In particular, the larger knowledge diversity inherent to complex technologies demands more diverse but specialized experts [54]; experts who must work together to solve complex problems [42].

**Fig 7:**
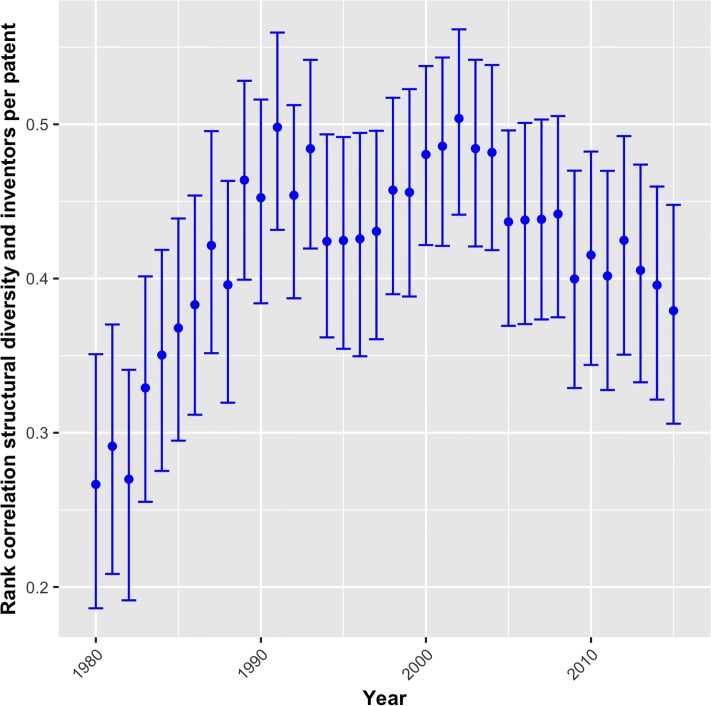
Correlation between *structural diversity* and the number of inventors per patent, 1980-2015.

Fig 7 shows the rank correlation between technologies’ *structural diversity* and the average number of inventors per patents. The latter signals the extent to which the patent is based on teamwork. The positive significant coefficient in all years underlines the importance of collaborative work in more complex technologies. While the correlation is relatively low in the beginning of the 1980s, it grows strongly, reaching a value of approximately 0.4 in the late 1980s. It remains somewhat above or close to this level in subsequent years. The finding clearly supports complex technologies involving more collaboration in R&D.

### 3.4 Complex technologies concentrate in space

In economic geography and regional science, it has long been argued that developing complex technologies requires special skills, existing expertise, infrastructure, and institutions not found everywhere [55–57]. Spatial proximity between experts is essential for face-to-face communication, which enhances work on complex projects [42]. The place-specificity of favorable conditions for (complex) innovation is also emphasized in concepts like the *learning regions*, *innovative milieu*, and *regional innovation systems* [58–60]. Such conditions allow for bridging cognitive distances and combining heterogeneous knowledge, which in other places would remain uncombined. These place-specificities are path-dependent and relatively rare. Consequently, complex technologies are argued to concentrate in space, which is supported by empirical evidence for the USA [3, 5].

**Fig 8:**
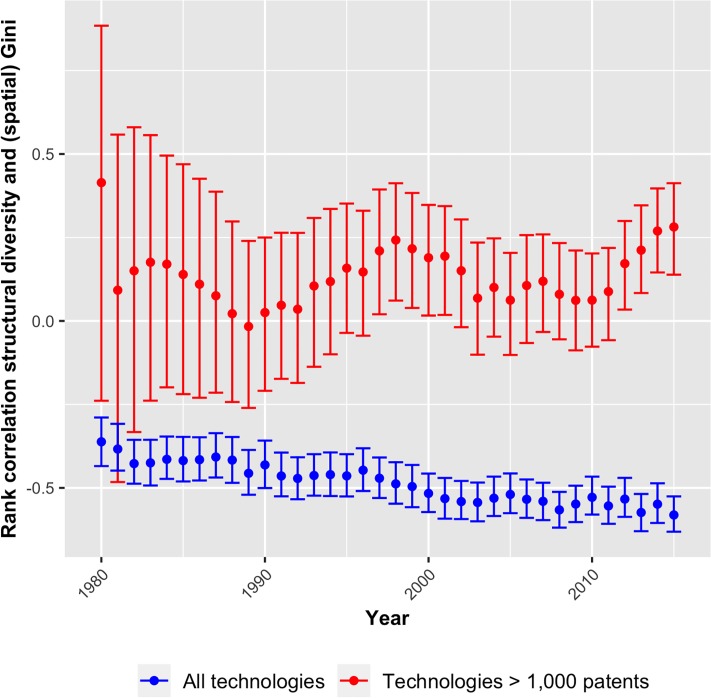
Correlation between *structural diversity* and technologies’ spatial concentration, 1980-2015.

To explore the relation between *structural diversity* and the spatial distribution of technologies, the residential information of patent inventors is used. More precise, for each NUTS2 region and technology, I count the number of patents with at least one of its inventors’ addresses being assigned to this region. Subsequently, Gini coefficients are estimated for the technology-specific regional distributions of patent counts (270 regions and 1, 557, 416 patents). These calculations are done with respect to European NUTS2 regions. Consequently, only patents are considered with at least one inventor from Europe. The coefficient obtains values close to one if inventors concentrate in a few regions and it converges to zero when they are evenly distributed in space. Fig 8 shows the correlation between technologies’ spatial Gini coefficients and their values of *structural diversity*. As technologies with few patents have less potential to be equally distributed across regions, the correlation is also presented for technologies with at least 1, 000 patents.

When considering all technologies (blue error bars), the correlations are strongly negative significant and suggest that complex technologies are more evenly distributed than simple technologies. However, the picture changes when concentrating on technologies with many patents (red error bars). Due to the small numbers of technologies with more than 1,000 patents, the correlations remain insignificant until the mid-1990s. From that year onward, the correlations fluctuate between being positive significant and insignificant. Notably, since 2009, the coefficient grows constantly and remains significant from 2012 onward, suggesting that larger and more complex technologies do increasingly concentrate in space. However, a similar trend was visible in the 1990s, which turned around in 1999. It is therefore not clear if it indicates systematic changes in the underlying processes or merely empirical fluctuations. Hence, the evidence is rather inconclusive with only the largest and most patent intensive technologies showing some spatial concentration in the most recent years.

### 3.5 Multivariate analysis

Thus far, the empirical analysis has analyzed differences between simple and complex technologies in a bivariate manner. This does not deliver insights into the relative importance of some of these differences or into the extent that they are related. Table 1 presents the results of a linear (within-estimator with time-fixed effects) panel regression with the *structural diversity* values of 646 technologies (those with at least five patents in two subsequent years) over the 36 years (1980-2015) as the dependent variable. All none-dummy explanatory variables are considered in logs, and robust clustered standard errors are used. As a robustness check, I repeat the analysis limiting the sample to the most recent 15 years. These results are reported in the supporting information (S3 Table). No substantial differences are observed between the two analyses. I therefore concentrate on the findings of the analysis based on all years in the following. The supporting information also includes an overview of the variables’ descriptives (S1 Table) and their correlations (S2 Table).

**Table 1:** Characteristics of *structural diversity*, 1980-2015.

	*Dependent variable:*							
	Structural complexity							
	(1)	(2)	(3)	(4)	(5)	(6)	(7)	(8)
Log(Patents)	1.003*** (0.036)	1.002*** (0.037)	0.913*** (0.039)	0.827*** (0.037)	0.897*** (0.053)		1.177*** (0.051)	0.903*** (0.038)
High		0.030 (0.252)	−0.148 (0.228)	−0.346* (0.197)	−0.390** (0.197)	0.842*** (0.325)		−0.132 (0.148)
Log(Median age + 1)			−2.324*** (0.266)	−2.032*** (0.239)	−1.977*** (0.239)			−1.083*** (0.182)
Log(Inventors per patent)				2.888*** (0.198)	2.837*** (0.197)			0.980*** (0.137)
Log(Spatial Gini)					2.000** (0.988)	−14.828*** (1.039)	5.195*** (1.102)	4.196*** (0.630)
log(CPCs per patent)								2.380*** (0.069)
adj. R2	0.396	0.396	0.421	0.485	0.486	0.177	0.404	0.653
n	646	646	646	646	646	646	646	646
T	36	36	36	36	36	36	36	36
N	22,360	22,360	22,360	22,360	22,344	22,344	22,344	22,310
Year fixed effects	Yes	Yes	Yes	Yes	Yes	Yes	Yes	Yes

Unbalanced panel regression, robust standard errors and p-values.

*p<0.1;

**p<0.05;

***p<0.01

The multivariate analyses confirm the previous bivariate results. More complex technologies tend to have more patents, which is underlined by the significantly positive coefficient of *Patents*. Hence, they are likely to require larger R&D efforts. More complex technologies are also younger, as the coefficient of *Median age* is significantly negative. The significantly positive coefficient of *Inventors per patent* confirms that R&D in complex technologies is conducted in a more collaborative fashion than in simple technologies. Accordingly, these characteristics of technological complexity are found to be true even when controlling for the respective others. Thus, to a significant extent, they characterize complex technologies independent of each other. This cannot be said about technologies being high-tech and their spatial distribution. The dummy for high technologies (*High-tech*) is strongly correlated to other explanatory variables, particularly *Median age* and *Patents*, which explains its insignificance in most models. The weakly significant negative coefficients of *High-tech* in Models (4) and (5) indicate that high technologies have relatively lower values of *structural diversity* when controlling for the average age of their patents, collaboration intensity, and patent numbers. When excluding these variables, the coefficient shows the expected significant positive sign. Accordingly, the larger complexity of high technologies seems to be primarily explained by these other features.

The measure of spatial concentration (*Spatial Gini*) also relates very strongly to the other explanatory variables in general and (negatively) to the number of patents in particular (see S2 Table in the supporting information). When controlling for the number of patents, it becomes significantly positive (Model 7). However, its significance is somewhat reduced when including the other explanatory variables (Model 5). Hence, patent numbers largely explain the observed negative bivariate relationship between *structural diversity* and technologies’ spatial concentration. More patents tend to make technologies more evenly distributed in space. Once this is accounted for, technologies with large values of *structural diversity* (complex technologies) are found to concentrate in space.

Lastly, to control for potential changes in the classification of patents to CPC classes, the number of CPC subclasses (10-digit) per patent (*CPCs per patent*) is added to the model. The variable becomes positive and significant in the model and primarily seems to lower the explanatory power of patents and technologies’ age. While the number of subclasses assigned to patents tends to positively correlated to *structural diversity*, all other results do not change substantially.

### 3.6 Comparison with two alternative measures of technological complexity

To put these results for the measure of *structural diversity* into perspective, I repeat the multivariate analysis for the two alternative approaches of quantifying technological complexity that have been or can be used in similar settings. The first is the complexity measure of *modular complexity* (*FS*.*modular*) introduced by *Fleming and Sorenson* [13]. In the present paper, it evaluates the frequency of patent subclass co-occurrences (10-digit CPC classes) on patents in a particular year (moving window of three years), in comparison with the cumulative frequency of their co-occurrences in all prior (to the moving window) years. The individual scores of patents are averaged (median) at the four-digit CPC level. Secondly, I follow *Balland and Rigby* [5] in calculating an index of technological complexity (*KCI*) based on technologies’ spatial distribution in year *t*. For this, the regional technological advantage (RTA) is calculated for all European regions (NUTS 2 and alternatively NUTS 3) and technologies (four-digit CPC). On this basis, a two-mode network between regions and technologies *T* is constructed with a binary link if region *r* has *RTA*_*r*,*T*,*t*_ > 1, i.e., when it is above average specialized in technology *T*. There is no link otherwise. The method of reflection with 20 iterations is applied to this network generating the complexity index *KCI* for year *t*. To resemble the construction of the *structural diversity* measure, the three-year moving window approach is employed in the construction of the annual patent data. The *KCI* is an index by construction, which requires the use of year-fixed effects in the regression to make it comparable across years. Moreover, it is advisable to transform it into ranks to control for annual variations in its variance. I estimate a regression for both versions, whereby log-transforming the ranks-based *KCI* substantially improves the model fit. However, the regression results obtained for the original and the ranks-based version of the *KCI* are identical in terms of the coefficients’ signs and significance.

The analysis (Table 2) reveals negative relations with R&D efforts (approximated by patents and belonging to high-tech) and collaborative R&D when approximating technologies’ complexity with the *KCI*. Older (*Median age*) and less collaborative technologies (*Inventors per patent*) are also found to score higher on this complexity index. A significantly positive relation is observed with spatial concentration (*Spatial Gini*). With exception of the latter, the results suggest this index to behave rather opposite to the stylized facts usually associated to technological complexity. This finding is independent of the chosen spatial unit (NUTS 2 or NUTS 3), which seems to have little relevance in this context.

**Table 2:** Characteristics of alternative measures of technological complexity, 1980-2015.

	*Dependent variable:*			
	Complexity			
	FS.Modular	HH.NUTS2	log(HH.NUTS2.rank)	HH.NUTS3
	(1)	(2)	(3)	(4)
log(Patents)	0.077*** (0.015)	−0.225* (0.118)	−0.074*** (0.021)	−0.367*** (0.095)
High-tech	0.115* (0.063)	−1.810*** (0.484)	−0.244*** (0.086)	−1.153*** (0.264)
Log(Median age)	0.262*** (0.065)	0.630 (0.581)	−0.115 (0.096)	0.406 (0.440)
Log(Inventors per patent)	0.107 (0.068)	−3.810*** (0.428)	−0.595*** (0.078)	−5.768*** (0.342)
Log(Spatial Gini)	2.553*** (0.305)	26.493*** (2.127)	4.711*** (0.392)	16.651*** (1.753)
adj. R2	0.062	0.125	0.208	0.154
n	646	646	646	646
T	36	36	36	36
N	21,974	22,355	22,355	22,355
Year fixed effects	Yes	Yes	Yes	Yes

Unbalanced panel regression, robust standard errors and p-values.

*p<0.1;

**p<0.05;

***p<0.01

In case of *FS*.*Modular*, the findings are somewhat more in line with these facts. The measure is found to be positively related to R&D efforts (*Patents*, *High-tech*), to collaborative R&D (*Inventors per patent*), and to the spatial concentration of patenting activities (*Spatial Gini*). However, similar to *KCI*, older technologies (*Median age*) are associated to higher levels of complexity, which contrasts the corresponding stylized fact.

This exercise is not intended and surely does not qualify as a fully developed comparison of the different approaches. However, it highlights the non-arbitrary character of choosing an indicator of technological complexity. Context matters, and, in some situations, certain features of an approach are desirable while in others they might be misleading. If it is important to have a measure reflecting the four stylized facts discussed above; of these three, *structural diversity* seems to mirror them most closely.

## 4 Summary & conclusion

Measuring the complexity of technologies has received significant attention from different disciplines. For instance, in engineering, technological complexity is argued to impact the costs and the management of technological systems [24]. Scholars use concepts and measures of technological complexity to better understand combinatorial R&D processes in innovation studies [13, 16]. Moreover, in economics and economic geography, technological complexity is seen as an important determinant of the uneven economic development in space [4, 5]. However, quantifying the complexity of technologies is literally a *complex* task and there is no widely accepted way to do it.

The present paper proposed a new measure of technological complexity called *structural diversity*, which approximates the diversity in how technologies’ (knowledge) subcomponents relate to each other. It was also argued that a slightly adapted version of *Emmert-Streib and Dehmer*’s *Network Diversity Score* [22] resembles this measure in empirical settings. Employing this approach, the study assessed the complexity of 655 patent classes (technologies) across 36 years. Co-occurrences of CPC subclasses on patents have been used to create technology-specific combinatorial networks, which, in turn, served as basis for the calculation of *structural diversity*. S4 Table in the supporting information provides the complete list of these technologies and their respective complexity values in the year 2014. The results for additional years are in the supplements.

Subsequently, it was shown that the obtained values mirror four stylized facts commonly associated with technological complexity: Complexity growth over time with complex technologies being on average younger. These technologies are also more R&D intensive and their R&D activities are more collaborative. When accounting for technologies with many patents being more widely distributed in space, complex technologies have moreover been shown to concentrate geographically.

The present study focused on the introduction of a new measure of technological complexity and explored its properties empirically. While the obtained results are promising, the empirical measurement of *structural diversity* calls for more work in the future. The application of the *Network Diversity Score* is an approximate measure of the diversity of network topologies. In addition, it is relatively computational intensive and requires the dichotomization of technologies’ combinatorial networks. The latter aspect particularly gives direction for future research, as it implies a significant loss of information contain in the patent data.

Further, the presented empirical analysis is only a first step towards a better understanding of the development of technological complexity over time and space as well as of its relation to socio-economic developments. For instance, when using this measure in future studies, it will be interesting to investigate the relevance of technological complexity for the economic growth of firms, regions, and countries. Similarly, evaluating the contribution of policy and public research to the advancement of simple and complex technologies will offer new insights into their over-all role in technological progress.

## Supporting information

S1 Fig Temporal correlation of *structural diversity*. (TIFF)

S2 Fig Share of main component. (TIFF)

S1 Table Descriptive statistics. (PDF)

S2 Table (Rank) correlation matrix. (PDF)

S3 Table Characteristics of *structural diversity*, 2001-2015. (PDF)

S4 Table Technologies and *structural diversity* in 2014. (PDF)

S5 Table Supplementary data. (XLSX)
