# Supplementary figures and images for: Using structural diversity to measure the complexity of technologies
Source: PLoS One. 2019 May 21;14(5):e0216856. doi: 10.1371/journal.pone.0216856 (PMC6528977; doi:10.1371/journal.pone.0216856)

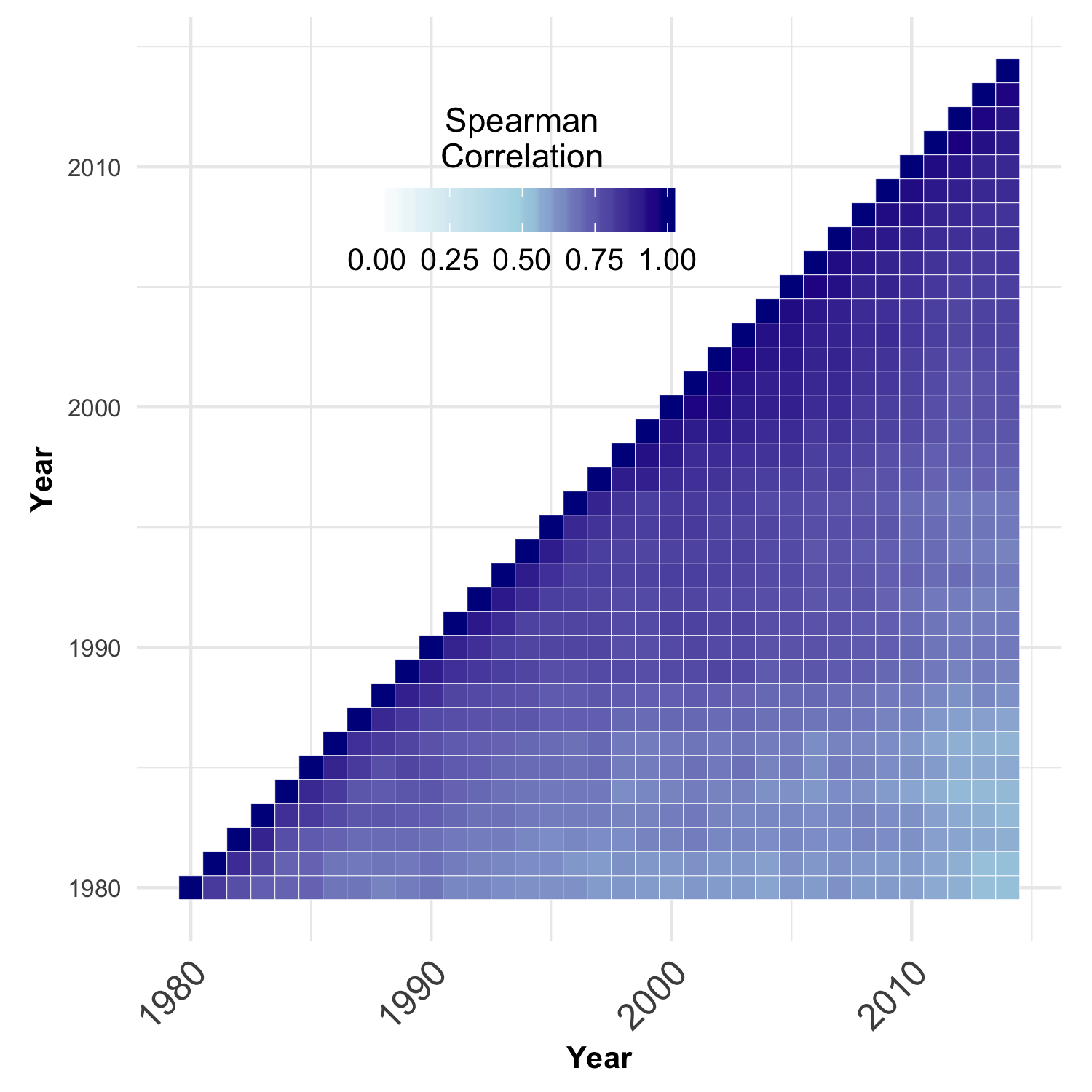

Supplement: S1 Fig — (TIFF) [file pone.0216856.s001.tiff]

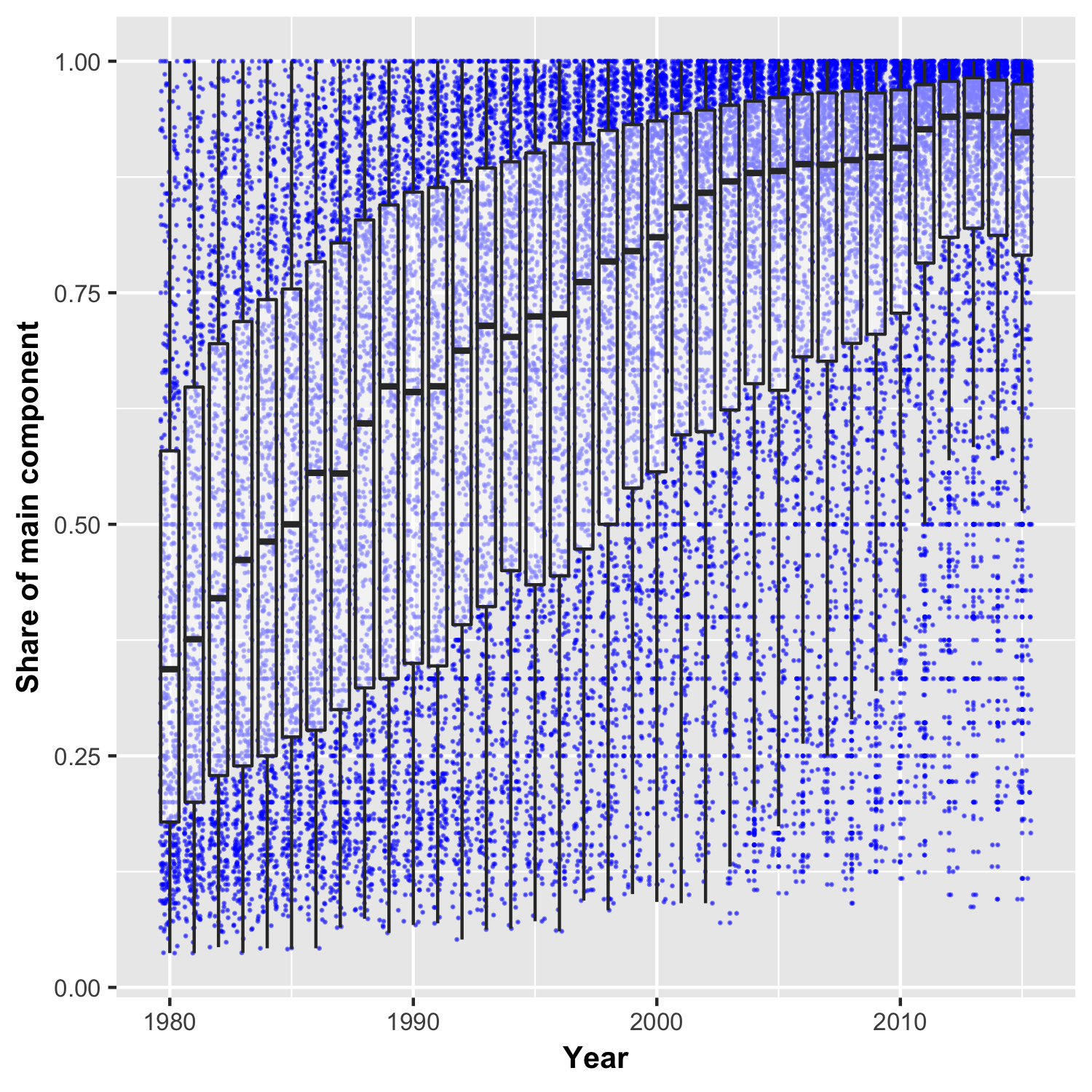

Supplement: S2 Fig — (TIFF) [file pone.0216856.s002.tiff]
