# Supplementary material for: Using structural diversity to measure the complexity of technologies
Source: PLoS One. 2019 May 21;14(5):e0216856. doi: 10.1371/journal.pone.0216856 (PMC6528977; doi:10.1371/journal.pone.0216856)
Supplement: S1 Table — (PDF) [file pone.0216856.s003.pdf]

S1 Table. Descriptive statistics of regression variables 1980-2015

Statistic	N	Mean	St. Dev.	Min	Pctl(25)	Pctl(75)	Max
Structural diversity	23,104	7.46	2.93	0.00	5.71	9.63	14.98
Patents	23,312	766.18	1,989.02	1.00	65.00	653.25	33,414.00
High-tech	23,580	0.04	0.21	0	0	0	1
Median age	23,473	6.66	3.62	0.00	3.76	9.13	25.34
Inventors per patent	23,312	1.95	0.51	0.50	1.59	2.25	12.00
Spatial Gini	23,190	0.90	0.06	0.69	0.86	0.95	1.00
CPCs per patent	22,932	4.64	2.83	1.00	3.00	6.00	68.00
FS.Modular	22,607	0.99	0.53	0.00	0.68	1.17	9.43
HH.NUTS2	23,312	88.02	17.17	0.00	77.00	99.98	100.00
