# Supplementary material for: Using structural diversity to measure the complexity of technologies
Source: PLoS One. 2019 May 21;14(5):e0216856. doi: 10.1371/journal.pone.0216856 (PMC6528977; doi:10.1371/journal.pone.0216856)
Supplement: S2 Table — (PDF) [file pone.0216856.s004.pdf]

S2 Table. (Rank) correlation Matrix of regression variables 1980-2015

	Structural	Patents	High tech	Median age	Inventors per patent	Spatial Gini	CPCs per patent	FS. Modular
Patents	0.66							
High-tech	0.07	0.09						
Median age	0.27	0.1	-0.05					
Inventors per patent	0.54	0.32	0.09	0.32				
Spatial Gini	-0.47	-0.8	0	-0.06	-0.15			
CPCs per patent	0.7	0.31	0.01	0.34	0.52	-0.24		
FS.Modular	-0.18	0.05	0.09	0.18	0.15	0.12	-0.4	
HH.NUTS2	-0.3	-0.21	-0.01	-0.49	-0.26	0.17	-0.29	-0.09
