# Supplementary material for: Using structural diversity to measure the complexity of technologies
Source: PLoS One. 2019 May 21;14(5):e0216856. doi: 10.1371/journal.pone.0216856 (PMC6528977; doi:10.1371/journal.pone.0216856)
Supplement: S3 Table — (PDF) [file pone.0216856.s005.pdf]

S3 Table. Characteristics of *structural diversity*, 2001-2015

	Dependent variable:							
	Structural diversity							
	(1)	(2)	(3)	(4)	(5)	(6)	(7)	(8)
Log(Patents)	0.969***	0.967***	0.821***	0.716***	0.757***		1.086***	0.749***
	(0.037)	(0.037)	(0.041)	(0.037)	(0.054)		(0.054)	(0.041)
High-tech		0.107	−0.011	−0.245	−0.266	0.966***		−0.048
		(0.249)	(0.237)	(0.215)	(0.215)	(0.300)		(0.178)
Log(Median age + 1)			−2.633***	−2.666***	−2.613***			−1.845***
			(0.289)	(0.255)	(0.258)			(0.217)
Log(Inventors per patent)				3.130***	3.095***			1.128***
				(0.205)	(0.205)			(0.169)
Log(Spatial Gini)					1.117	−14.831***	3.433***	2.671***
					(1.013)	(1.029)	(1.142)	(0.690)
Log(CPCs per patent)								2.130***
								(0.083)
adj. R2	0.456	0.456	0.498	0.577	0.575	0.233	0.459	0.702
n	643	643	643	643	643	643	643	643
T	15	15	15	15	15	15	15	15
N	9,433	9,433	9,433	9,433	9,425	9,425	9,425	9,407
Year fixed effects	Yes	Yes	Yes	Yes	Yes	Yes	Yes	Yes

Unbalanced panel regression, robust standard errors and p-values. *p<0.1; **p<0.05; ***p<0.01
