# Supplementary material for: Using structural diversity to measure the complexity of technologies
Source: PLoS One. 2019 May 21;14(5):e0216856. doi: 10.1371/journal.pone.0216856 (PMC6528977; doi:10.1371/journal.pone.0216856)
Supplement: S4 Table — (PDF) [file pone.0216856.s006.pdf]

S4 Table. Technologies and *structural diversity* in 2014

Rank	CPC	Patents	*Structural diversity*	Rank	CPC	Patents	*Structural diversity*	Rank	CPC	Patents	*Structural diversity*
1	B60L	3,098	13.356	61	B60K	3,438	11.782	121	F03B	488	11.175
2	Y04S	1,233	13.227	62	D04H	747	11.766	122	H02P	1,881	11.174
3	H04W	22,610	12.922	63	B60M	176	11.760	123	A43B	886	11.171
4	B60W	2,551	12.731	64	A23V	2,263	11.725	124	H04R	2,791	11.164
5	C10N	788	12.668	65	B42D	684	11.725	125	C25D	985	11.157
6	F17C	706	12.661	66	C10G	1,392	11.704	126	C08L	6,697	11.143
7	H03F	826	12.631	67	B23K	3,419	11.699	127	G06Q	8,663	11.143
8	Y02C	614	12.617	68	F05B	1,905	11.696	128	B29B	1,078	11.136
9	B33Y	1,211	12.566	69	C12Y	2,347	11.684	129	H01G	1,152	11.134
10	A61H	926	12.518	70	H04N	13,841	11.670	130	H05K	4,911	11.116
11	C21D	1,607	12.516	71	B29L	4,310	11.631	131	B60B	698	11.108
12	F02D	2,213	12.504	72	H04S	769	11.612	132	F02B	1,833	11.106
13	F01N	2,222	12.454	73	B21B	548	11.611	133	F01L	691	11.075
14	C01P	1,504	12.427	74	C10L	948	11.606	134	C12M	1,281	11.067
15	C12Q	4,428	12.413	75	F21W	624	11.600	135	E05Y	1,315	11.060
16	F21K	1,033	12.396	76	C05F	232	11.596	136	B41M	1,021	11.060
17	F05D	5,581	12.389	77	C11D	1,720	11.553	137	F23G	303	11.059
18	H02M	3,240	12.362	78	C12P	2,419	11.546	138	A61N	3,343	11.058
19	F01D	5,568	12.349	79	C08J	4,045	11.528	139	B44C	383	11.018
20	F21S	1,957	12.341	80	C13K	172	11.526	140	A23C	497	11.018
21	B22F	1,662	12.286	81	G10L	2,090	11.514	141	H04Q	1,051	11.003
22	A61Q	3,657	12.282	82	C22F	599	11.512	142	C09B	607	11.002
23	G06T	7,184	12.271	83	A23Y	154	11.493	143	A61L	3,766	10.995
24	C01G	768	12.267	84	A43C	249	11.491	144	A21D	333	10.980
25	H02J	5,532	12.256	85	D06N	228	11.481	145	C01F	393	10.966
26	B32B	6,486	12.254	86	B29K	4,060	11.479	146	G07C	1,416	10.956
27	C09C	583	12.222	87	B82Y	1,555	11.479	147	G06K	6,900	10.950
28	C10M	917	12.208	88	D03D	473	11.451	148	F28D	2,030	10.917
29	F21Y	2,240	12.175	89	A23D	287	11.443	149	G01C	2,759	10.901
30	G09G	2,743	12.171	90	A61B	17,651	11.438	150	H04H	577	10.900
31	D07B	234	12.166	91	F25B	2,098	11.434	151	E04F	1,145	10.889
32	B01L	1,941	12.159	92	B29C	8,927	11.425	152	E05F	999	10.883
33	C10J	262	12.139	93	F24J	861	11.423	153	H01Q	2,440	10.867
34	A61K	22,848	12.133	94	A01N	2,830	11.413	154	D10B	839	10.867
35	C22C	3,006	12.122	95	C08K	5,155	11.384	155	C08G	5,480	10.860
36	F23R	1,040	12.078	96	B60G	940	11.371	156	C07B	828	10.854
37	C10K	226	12.049	97	C25B	707	11.365	157	B60Q	1,176	10.845
38	H01M	7,041	12.041	98	F04D	2,630	11.346	158	H04B	8,740	10.843
39	A63B	1,276	12.007	99	H01S	980	11.331	159	C09D	5,282	10.842
40	F03D	2,108	11.996	100	A23P	431	11.330	160	C21B	218	10.835
41	C07K	7,229	11.980	101	C10B	281	11.323	161	B05D	1,878	10.823
42	C09J	2,366	11.972	102	D21H	1,074	11.309	162	A45D	1,080	10.817
43	C07D	8,552	11.965	103	G08C	706	11.302	163	A24F	742	10.817
44	B60Y	1,263	11.958	104	F02P	335	11.298	164	G06F	32,551	10.811
45	H04J	1,979	11.954	105	Y02T	10,645	11.283	165	A23G	670	10.803
46	H04L	27,170	11.921	106	C11B	461	11.274	166	C12R	320	10.801
47	A23K	757	11.913	107	H04M	4,799	11.268	167	G02C	972	10.799
48	F02N	490	11.908	108	F23J	340	11.268	168	D01D	460	10.798
49	H01L	12,946	11.907	109	B62M	588	11.258	169	G06N	1,121	10.793
50	F25J	319	11.901	110	F15B	1,187	11.246	170	F02M	2,530	10.790
51	C02F	2,293	11.871	111	C07C	5,460	11.244	171	C01B	2,858	10.783
52	E02F	1,157	11.856	112	A61M	8,004	11.231	172	B60C	2,324	10.780
53	F02C	2,991	11.852	113	F15C	8	11.205	173	C30B	565	10.765
54	C04B	2,787	11.849	114	B22D	977	11.200	174	H02K	3,912	10.765
55	F21V	2,975	11.827	115	B61C	165	11.194	175	D04B	464	10.734
56	F02K	880	11.813	116	B28B	584	11.183	176	B62K	978	10.724
57	B61L	499	11.812	117	B01J	5,367	11.181	177	F28F	1,958	10.724
58	A23L	3,142	11.793	118	B60T	1,665	11.179	178	A23J	194	10.723
59	C12N	8,072	11.792	119	F01K	868	11.178	179	H02H	1,323	10.709
60	G08G	1,745	11.787	120	G03H	194	11.176	180	E05D	921	10.703

Rank	CPC	Patents	*Structural diversity*	Rank	CPC	Patents	*Structural diversity*	Rank	CPC	Patents	*Structural diversity*
181	A01H	331	10.700	242	C07H	810	10.190	303	C22B	575	9.736
182	F21L	117	10.683	243	F16C	2,624	10.187	304	B64D	2,669	9.734
183	B06B	468	10.682	244	B29D	2,066	10.184	305	B23P	1,458	9.730
184	F24D	891	10.682	245	F04B	1,847	10.183	306	A63F	812	9.704
185	B31B	442	10.627	246	C05B	103	10.173	307	A46D	119	9.703
186	D21C	340	10.621	247	G03F	1,097	10.157	308	F16L	3,062	9.699
187	E04C	842	10.618	248	A41D	745	10.139	309	B25F	548	9.663
188	G04G	280	10.616	249	A47L	1,911	10.135	310	E05C	587	9.660
189	G01J	1,550	10.614	250	A61F	6,780	10.134	311	B61H	94	9.659
190	C08C	267	10.609	251	B25J	1,585	10.125	312	G05G	404	9.636
191	G11C	1,104	10.583	252	A45F	246	10.121	313	C09K	3,399	9.635
192	A23F	248	10.573	253	B27D	99	10.121	314	B24D	301	9.635
193	G09C	262	10.553	254	B41C	143	10.113	315	B21C	392	9.626
194	F04C	1,615	10.548	255	F25C	192	10.103	316	E04D	502	9.620
195	G07D	605	10.532	256	G01T	702	10.095	317	F16D	2,659	9.614
196	F01C	345	10.531	257	G08B	1,970	10.090	318	A61G	1,017	9.612
197	E05B	1,576	10.529	258	A45C	434	10.082	319	B23C	481	9.601
198	G03B	1,475	10.524	259	B03C	310	10.077	320	H04K	99	9.600
199	C21C	267	10.520	260	G01S	4,267	10.072	321	H03L	306	9.566
200	F23L	250	10.513	261	C05G	201	10.056	322	H01P	550	9.557
201	F01P	492	10.504	262	C05C	102	10.042	323	B42C	106	9.546
202	D06F	1,805	10.492	263	G05B	4,570	10.024	324	F23M	186	9.524
203	F24F	2,029	10.491	264	B60R	3,738	10.023	325	A24D	362	9.518
204	F16M	822	10.475	265	A41B	176	10.012	326	B65H	1,930	9.511
205	H03G	406	10.467	266	H01F	2,431	9.992	327	B62B	472	9.509
206	A61C	1,588	10.449	267	B27K	96	9.989	328	B65F	245	9.508
207	C08H	248	10.431	268	C03B	807	9.987	329	B03D	100	9.496
208	D01F	696	10.430	269	Y02E	11,568	9.980	330	B60H	793	9.492
209	B64C	2,314	10.420	270	D05C	57	9.978	331	B63G	189	9.485
210	D02G	287	10.416	271	F23D	610	9.962	332	C05D	130	9.484
211	C07J	192	10.383	272	B26D	813	9.959	333	G11B	1,080	9.474
212	C07F	2,073	10.383	273	G07F	1,022	9.946	334	F16B	2,539	9.473
213	B04B	239	10.382	274	B21J	380	9.943	335	F23C	414	9.469
214	G02F	2,326	10.382	275	G06M	47	9.935	336	H02N	386	9.449
215	B04C	126	10.374	276	B81C	386	9.929	337	C40B	115	9.449
216	H01R	3,558	10.374	277	C12C	128	9.926	338	B01F	1,588	9.448
217	B81B	432	10.362	278	G01N	15,372	9.926	339	B64G	313	9.441
218	F25D	1,619	10.360	279	H05B	4,271	9.921	340	D21B	88	9.439
219	B01D	6,664	10.359	280	Y02B	7,205	9.917	341	G07B	278	9.435
220	A46B	541	10.355	281	E21B	3,963	9.913	342	G01B	2,531	9.425
221	H03M	1,123	10.336	282	E04B	1,625	9.905	343	B64F	500	9.421
222	G09B	1,095	10.317	283	A61J	1,091	9.887	344	Y02P	6,950	9.416
223	B65B	2,495	10.309	284	C07G	91	9.884	345	C23G	151	9.415
224	C23C	3,703	10.295	285	G09F	879	9.877	346	B62J	661	9.408
225	A23B	312	10.276	286	F27D	713	9.876	347	C01D	126	9.404
226	C03C	1,468	10.271	287	G10K	759	9.867	348	F01M	474	9.403
227	H01B	2,038	10.269	288	B27N	132	9.854	349	G21D	156	9.400
228	B25D	242	10.268	289	G01V	2,028	9.852	350	B23H	195	9.398
229	G10H	258	10.264	290	G01R	4,554	9.829	351	F05C	149	9.388
230	B41J	2,388	10.260	291	B67C	330	9.824	352	Y10T	17,313	9.388
231	H02S	755	10.254	292	G07G	175	9.811	353	D04C	94	9.380
232	Y02W	1,474	10.248	293	H05H	475	9.805	354	A01D	1,027	9.374
233	C08F	3,794	10.243	294	F27B	543	9.802	355	B63H	537	9.371
234	F23N	406	10.240	295	G05F	575	9.801	356	E03C	472	9.352
235	C08B	678	10.239	296	G02B	9,406	9.795	357	C01C	152	9.313
236	F24H	656	10.235	297	F16H	3,579	9.781	358	H01J	1,914	9.306
237	B09B	263	10.229	298	B65D	5,994	9.770	359	G21F	319	9.297
238	A24B	290	10.222	299	G21K	347	9.760	360	F03G	370	9.288
239	D06M	641	10.211	300	F02G	181	9.758	361	D06P	212	9.283
240	G21C	449	10.206	301	F16N	249	9.742	362	B08B	1,083	9.278
241	B60N	1,353	10.199	302	A43D	139	9.740	363	B67D	583	9.271

Rank	CPC	Patents	*Structural diversity*	Rank	CPC	Patents	*Structural diversity*	Rank	CPC	Patents	*Structural diversity*
364	H05G	168	9.268	425	F16J	1,351	8.708	486	G01Q	113	8.092
365	B09C	91	9.267	426	A47C	973	8.704	487	G01G	454	8.091
366	B62D	3,054	9.250	427	B07B	243	8.686	488	C23D	25	8.080
367	B61K	96	9.238	428	F01B	171	8.682	489	F27M	12	8.064
368	H01C	260	9.219	429	B68F	2	8.675	490	G04R	66	8.062
369	B42B	52	9.218	430	A21C	182	8.664	491	B25C	196	8.060
370	B23B	976	9.218	431	G21Y	45	8.645	492	E05G	61	8.042
371	B66B	826	9.216	432	B62H	127	8.643	493	B66C	742	8.040
372	C12G	114	9.215	433	C25F	111	8.635	494	E03D	326	8.030
373	B02C	759	9.185	434	A62B	467	8.630	495	A45B	95	8.026
374	B43K	138	9.182	435	F24C	1,079	8.628	496	A44C	251	7.996
375	G01P	892	9.176	436	B24C	211	8.626	497	H02B	462	7.988
376	C25C	179	9.166	437	B21D	1,393	8.591	498	A41H	47	7.983
377	A42B	294	9.155	438	B63J	151	8.588	499	D21F	321	7.981
378	B22C	374	9.145	439	A01C	514	8.571	500	A47F	528	7.980
379	B60D	261	9.134	440	A63C	303	8.565	501	B25B	857	7.969
380	F22B	456	9.115	441	F23B	92	8.548	502	F28C	72	7.912
381	B62L	105	9.113	442	B65G	2,574	8.543	503	A23N	150	7.910
382	B82B	38	9.112	443	F28B	71	8.541	504	H03C	43	7.904
383	E06B	1,811	9.107	444	B60J	803	8.529	505	A47G	638	7.890
384	B05B	2,012	9.101	445	B44B	66	8.527	506	C06B	109	7.866
385	F02F	467	9.088	446	F26B	560	8.512	507	F41C	91	7.866
386	E02B	403	9.056	447	F41G	283	8.501	508	E01C	566	7.852
387	B65C	286	9.046	448	D02J	68	8.492	509	F42B	447	7.848
388	F23K	189	9.030	449	G01H	370	8.483	510	B21H	97	7.844
389	A44B	308	9.011	450	A61D	173	8.470	511	A01F	438	7.841
390	A01K	1,497	8.998	451	G01F	1,661	8.462	512	F16G	336	7.835
391	B26F	316	8.980	452	B60S	643	8.446	513	F28G	80	7.798
392	H03K	1,756	8.961	453	A41F	101	8.441	514	A47D	135	7.783
393	F16K	2,763	8.949	454	F15D	128	8.438	515	A63H	344	7.735
394	A22C	351	8.947	455	E21C	172	8.428	516	D01G	138	7.733
395	B64B	47	8.937	456	G01W	118	8.406	517	B01B	18	7.718
396	Y10S	1,752	8.934	457	A01B	606	8.404	518	B66D	223	7.716
397	A47J	2,161	8.933	458	D06B	127	8.404	519	F16P	168	7.708
398	G01K	917	8.911	459	D06C	100	8.356	520	B60P	593	7.707
399	C09G	126	8.908	460	H03D	116	8.338	521	B28D	303	7.703
400	D06Q	25	8.895	461	B31D	128	8.333	522	F41H	416	7.689
401	B05C	713	8.883	462	E04H	1,033	8.331	523	A01M	395	7.660
402	G05D	2,300	8.871	463	E02D	689	8.329	524	A63G	124	7.655
403	A44D	32	8.870	464	B21K	249	8.321	525	B23D	583	7.630
404	B27M	105	8.866	465	B28C	97	8.319	526	F22G	47	7.590
405	H01H	2,499	8.863	466	C11C	198	8.317	527	F23Q	144	7.585
406	H01T	315	8.839	467	A01J	155	8.302	528	B41K	41	7.576
407	H02G	1,550	8.815	468	F16F	1,767	8.301	529	G21B	61	7.574
408	B41F	682	8.801	469	G01M	2,017	8.259	530	D01H	235	7.572
409	B44F	96	8.796	470	B68G	49	8.257	531	F22D	53	7.549
410	G04B	654	8.782	471	C12F	27	8.244	532	F24B	92	7.547
411	C23F	368	8.761	472	C10C	31	8.242	533	E03B	195	7.528
412	F03C	105	8.754	473	A47K	683	8.231	534	B24B	856	7.509
413	B07C	297	8.748	474	B23F	126	8.227	535	E01D	124	7.507
414	F17D	191	8.742	475	A41C	92	8.218	536	B61F	192	7.460
415	F42D	89	8.741	476	A47B	1,183	8.208	537	A24C	279	7.393
416	A62D	110	8.739	477	H03B	140	8.191	538	D01B	8	7.367
417	G01D	1,931	8.736	478	G06E	17	8.173	539	G04C	171	7.321
418	B41P	175	8.735	479	G10G	28	8.164	540	E03F	324	7.318
419	H03H	508	8.735	480	B30B	534	8.162	541	A21B	137	7.316
420	B66F	599	8.732	481	G01L	1,553	8.146	542	B43M	29	7.316
421	G03G	1,548	8.730	482	B61D	440	8.146	543	B25G	131	7.288
422	A62C	454	8.725	483	B41N	137	8.135	544	F04F	156	7.276
423	B03B	91	8.717	484	B23Q	1,019	8.114	545	E04G	570	7.257
424	B63B	1,227	8.709	485	B31F	187	8.096	546	A01G	1,027	7.251

Rank	CPC	Patents	*Structural diversity*	Rank	CPC	Patents	*Structural diversity*	Rank	CPC	Patents	*Structural diversity*
547	B21F	148	7.241	584	F41F	42	6.514	621	G10F	15	4.356
548	F03H	30	7.227	585	F42C	97	6.406	622	A42C	18	4.141
549	C13B	38	7.215	586	B67B	136	6.350	623	A63D	17	4.079
550	D05B	130	7.183	587	B27G	84	6.336	624	G12B	10	4.042
551	G21G	61	7.141	588	B60F	37	6.268	625	B42P	19	3.794
552	D21D	76	7.111	589	E06C	102	6.193	626	B68C	33	3.329
553	B42F	45	7.100	590	C06D	47	6.137	627	B60V	10	3.156
554	C14C	44	7.099	591	C14B	50	6.134	628	B68B	13	3.064
555	C12H	58	7.064	592	F23H	26	6.122	629	H01K	28	2.682
556	B61B	147	7.039	593	D03C	29	6.108	630	G09D	2	2.679
557	B44D	40	7.022	594	E01B	290	6.069	631	C12J	5	2.209
558	E01F	284	7.018	595	B31C	18	6.033	632	H05C	6	2.123
559	D21J	32	7.009	596	E21D	154	5.993	633	B02B	5	1.171
560	H03J	54	6.992	597	G04D	65	5.973	634	B41G	2	0
561	B63C	201	6.980	598	A22B	83	5.933	635	B61J	8	0
562	F41A	351	6.956	599	B27C	41	5.917	636	C06F	2	0
563	B27B	262	6.902	600	E21F	58	5.901	637	C09H	1	0
564	D06H	53	6.895	601	B21G	11	5.813	638	C10F	2	0
565	G06G	27	6.889	602	B27L	40	5.785	639	C10H	2	0
566	D21G	117	6.877	603	A41G	40	5.778	640	C12L	1	0
567	B41D	9	6.851	604	D06J	2	5.760	641	D04G	5	0
568	B27J	5	6.849	605	A63J	68	5.595	642	D05D	11	0
569	B43L	45	6.837	606	H05F	38	5.537	643	D06G	3	0
570	A47H	64	6.830	607	B27H	2	5.326	644	E02C	1	0
571	D06L	44	6.810	608	A01L	30	5.280	645	G06C	2	0
572	D01C	18	6.806	609	F16S	9	5.260	646	G06J	1	0
573	G03C	31	6.805	610	B25H	201	5.226	647	B41B	–	–
574	G04F	141	6.754	611	F41J	53	5.214	648	B62C	–	–
575	D03J	25	6.729	612	D04D	12	5.182	649	F17B	–	–
576	C09F	23	6.706	613	F16T	21	5.090	650	F21H	–	–
577	B26B	406	6.657	614	G10D	111	5.008	651	G03D	–	–
578	F41B	84	6.622	615	E01H	148	4.986	652	G06D	–	–
579	B61G	68	6.595	616	G21H	14	4.957	653	G10B	1	–
580	B23G	49	6.587	617	A63K	14	4.723	654	G21J	–	–
581	B41L	10	6.570	618	B27F	28	4.623	655	H04T	–	–
582	C06C	26	6.563	619	D02H	6	4.513				
583	G10C	33	6.526	620	B21L	14	4.431				
